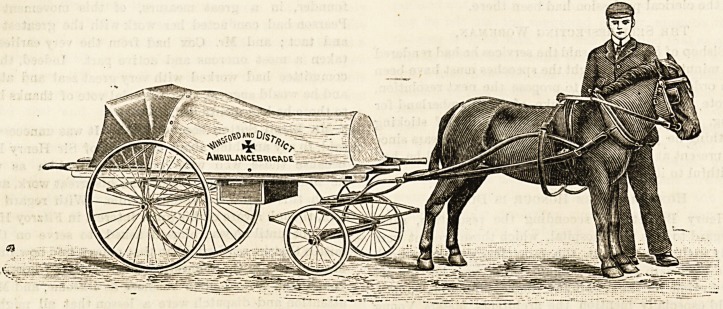# Practical Departments

**Published:** 1903-01-17

**Authors:** 


### PRACTICAL DEPARTMENTS.

### A LIGHT AMBULANCE LITTER.

For country districts, or places where it is not practicable or necessary to afford a horse ambulance, the light litter shown in our illustration should be of great use. It may be drawn either by a pony or by hand, and the makers supply a handle, as well as a pair of shafts, with each ambulance. These are interchangeable. The litter runs easily, and, being on four wheels, is both steady and easy to turn. The cover is of rubber sheeting, and the hood can be used or thrown back as required. There is a drawer for splints, bandages, etc., and the carriage runs on rubber-tyred wheels. Used as a bier, with the stretcher removed, it is suggested that it would be much more pleasing than the usual heavy and gloomy-looking hearse, and especially suitable for country funerals, and this use is specially noted by the makers, Messrs. Wilson and Stockall, of Bury, Lancashire. We cannot, however, altogether approve of such a combination of uses. Sentiment goes for something, and we think many people might justly object. Such an ambulance has just been supplied by them to Winsford, a small country town in Cheshire.

## Figures and Tables

**Figure f1:**